# Seroprevalence of Anti-*Toxoplasma gondii* Antibodies Among Pregnant Woman in South Khuzestan, Iran

**DOI:** 10.5812/jjm.9998

**Published:** 2014-05-01

**Authors:** Mohammad Jafar Yad Yad, Nabi Jomehzadeh, Maryam Jafar Sameri, Nooshin Noorshahi

**Affiliations:** 1 Infectious Disease and Tropical Medicine Research Center, Ahvaz Jundishapur University of Medical Sciences, Ahvaz, IR Iran; 2 Laboratory of Taleghani Hospital, Abadan School of Medical Sciences, Abadan, IR Iran; 3 Department of Obstetrics and Gynecology of Taleghani Hospital, Abadan School of Medical sciences, Abadan, IR Iran; 4 Laboratory of Arvand Medical Science University, International Branch of Jundishapur Medical Sciences University, Abadan, IR Iran

**Keywords:** Toxoplasmosis, Congenital, Pregnant Women, Seroepidemiologic Studies

## Abstract

**Background:**

Acute toxoplasmosis may lead to congenital toxoplasmosis with fetal complications outcome during pregnancy. Anti-* Toxoplasma gondii *antibody seroprevalence is unclear in pregnant women of south of Khuzestan province, since limited data about *T. gondii* seroepidemiology has been published in pregnant women of this area (Abadan, Shadegan, Khoramshar).

**Objectives:**

The aim of this study was to clarify the status of *T. gondii* seroprevalence in pregnant women of south of Khuzestan province.

**Materials and Methods:**

In this cross-sectional study, 501 full-term pregnant women were included. This study was carried out in Taleghani teaching hospital for six 6 months from May to October 2011. Informed consents signed by the patients were obtained. Blood IgG and IgM were measured using ELISA technique. The data was analyzed by SPSS 13 (Chicago, IL, USA). Chi-square test was used for comparison.

**Results:**

The participants’ age range was 15 to 45 years (average: 27.4 ± 13). Of the 501 pregnant women, 70.65 % (n = 354) were seronegative for *T. gondii* IgG and IgM antibodies. There were statistical relationships between IgG seroprevalence and age, as well as IgG seroprevalence and cat holding.

**Conclusions:**

There was high percentage of seronegative (70.65 %) IgG and IgM antibodies in full-term pregnant women. They were susceptible to acute toxoplasmosis; thus, prenatal screening was recommend in our province after cost-beneficial analyses.

## 1. Background

*Toxoplasma gondii* is an obligatory intracellular parasite, ian agent of zoonotic diseases. This parasite has a worldwide distribution. The most common routes of transmission in humans are through eating raw or half-cooked meat infected with tissue cysts, oocyst presence in contaminated water and vegetables, and mother-to-fetus vertical transmission (1). Acute infection in immunocompetent patients is benign, often self-limiting, and usually asymptomatic. The most common clinical manifestation of acute infection is cervical lymphadenitis. 

Chronic infection before pregnancy does not cause transmission to the fetus, but acute infections in untreated pregnant women may cause fetal transmission and congenital toxoplasmosis with fetal complications outcome (2). Clinical presentations of congenital toxoplasmosis vary. These can include chorioretinitis, blindness, mental retardation, pneumonias and encephalitis (1, 2). Seroprevalence of *T. gondii *was reported In America, Paris, Colombia and Austria, 31.7%, 83%, 45.8% and 35% among pregnant women, respectively (2-5).

In a similar study in Nigeria, seroprevalence rates of toxoplasmosis were estimated to vary from 7% - 51.3% in normal pregnant women to 17.5% - 52.3% in women with abnormal pregnancies and abortions (6, 7). Conducted studies in Senegal and Sudan in pregnant women showed IgG seroprevalence rates of 40.2% and 34.1% (8, 9). Many studies have been conducted about seroprevalence of *T. gondii *among pregnant women in different region of Iran, e.g. Kashan, Khorram-Abad, Tabriz, with IgG seroprevalence rates of 50.8%, 31% and 21.6%, respectively (10-12). Limited data have been published about pregnant women of south of Khuzestan province (Abadan, Shadegan, Khorramshahr).

## 2. Objectives

The aim of this study was to clarify the seroprevalence of *T. gondii* among pregnant women of south of Khuzestan province.

## 3. Materials and Methods

This cross-sectional study was conducted on 501 full-term pregnant women, aged 14-45 years, with no history of congenital or acquired immunodeficiency or diabetes in Taleghani teaching hospital, (patients population coverage of over 500000), for 6 months from May, to October 2011. The demographic data were recorded (age, cat holding, urban or rural residence). After taking the informed consents, a trained person distributed the questionnaires. Blood samples of 5 mL were taken from the participants by aseptic technique. After 10 minutes of 2000 rpm centrifugation, serum samples were kept at 20°C until assayed. All the samples were tested by ELISA for detection of *T. gondii* IgG and IgM antibodies using ELISA kit (Omega, England). Data were analyzed by SPSS 13 (Chicago, IL, USA). Chi-square test was used for comparison. Ethics Committee of Ahvaz Jundishapur University of Medical Sciences approved this research.

## 4. Results

In this study, 501 full-term pregnant women with an average age of 27.4 ± 5.4 years participated. Among them, 354 (70.65%) were seronegative for IgG and IgM antibodies, while 137 cases (27.3%) were positive for IgG and 7 (1.39%) for IgM (Table 1). There was not a statistical relationship between IgG and IgM antibodies and rural or urban residency (P value < 0.5 and P value = 0.06, respectively) (Table 2). Statistical relationship between cat holding and presence of IgG (P value < 0.001) as well as age and the presence of IgG (P value < 0.001) were significant (Table 3).

**Table 1.** Seroprevalence of *T. gondii* IgM and IgG According to the Age Group ^a^

Age Group	IgG (+)	IgM (+)	IgG and IgM (+)	IgG and IgM (-)	Total
**15-20**	17 (3.3)	-	-	28 (5.5)	45 (8.8)
**21-25**	28 (5.5)	-	-	148 (29.5)	176 (35.1)
**26-30**	45 (8.8)	6 (1.1)	3 (0.5)	80 (15.9)	134 (26.7)
**31-35**	32 (6.2)	1 (0.2)	-	63 (12.5)	96 (19.1)
**36-40**	14 (2.7)	-	-	18 (3.5)	32 (6.2)
**41-45**	1 (0.2)	-	-	17 (3.3)	18 (3.5)
**Total**	137 (27.3)	7 (1.3)	3 (0.5)	354 (70.6)	501

^a^ Data are presented in No. (%).

**Table 2.** Seroprevalence of Anti *T. gondii* IgG and IgM According to the Residency Area ^a^

Area	IgG (+) IgM (-)	P Value	IgG (-) IgM (+)	P Value	Total
**Rural**	128 (25.5)	< 0.5	4 (0.7)	< 0.06	431 (86)
**Urban**	7 (1.3)	< 0.5	-	-	70 (13.9)
**Total**	135 (100)		4		501

^a^ Data are presented in No. (%).

**Table 3.** Relationship of Anti- *T. gondii *IgG and IgM With Cat Holding ^a^

	IgG (+)	IgM (+)	P Value
**Cat holding**	34 (57.6)	1 (1.8)	< 0.001

^a^ Data are presented in No. (%).

## 5. Discussion

Seroprevalence of *T. gondii* varies in different regions of the world (1). The seroprevalence in women of child-bearing age in USA, Brazil, Argentina and Colombia was 11.0%, 7.3–77.5%, 48.7–53.4%, and 47.0-63.5%, respectively, while in Europe it varied between 8.2% (in Switzerland) and 63.2% (in Western Pomerania, Germany). In Asia and Oceania, the seroprevalence ranged from 0.8% (Suwon region, South Korea) to 63.9% (Babol, Iran) and in Africa, it was between 25.3% (Burkina Faso) and 75.2% (Sao Tomeand Principe) (13). 

In many studies conducted in different regions of Iran, *T. gondii* seroprevalence rate varied, e.g. Meshkinshahr, Yazd, Kermanshah, Karaj, Saveh and Islamshahr, in which, prevalence rates of IgG antibody in pregnant women were 21.8%, 39.8%, 36.3%, 45, 35.5% and 39%, respectively (14-18). The highest seroprevalence rate of IgG anti *T. gondii* antibody was reported in north of Iran (19). In Sari and Amole cities, 76.4% and 75.7% of women possessed seropositive IgG anti-*T. gondii* antibody, respectively (20). In the present study, seroprevalence of IgG anti *T. gondii* was 27.3%. Therefore, compared to north of Iran, seroprevalence of IgG anti *T. gondii* in south of Khuzestan province was low.

Seroprevalence of *T. gondii* was shown to increase with age (1, 3, 21, 22). This study also found the effect of age on seroprevalence of *T. gondii *among pregnant women. The statistical relationship between age and presence of IgG anti-*T. gondii* antibody (P value < 0.001) was significant (Table 3). In addition, IgG seroprevalence rate increased significantly with age, from 16% in 21-25-year age group to 44% in 36-40-year one (Table 1). Epidemiological survey has revealed that in most areas of the world, presence of cats is the primary important reason for the parasite transmission (1, 23, 24). 

In this study, the statistical relationship between cat holding and presence of IgG anti-*T. gondii* (P value < 0.001) was significant (Table 3). Some studies conducted in other regions of Iran, e.g. Ardebil, Kamyaran and Khorram-Abad, did not show same statistical relationships (11, 25, 26). In this study, 291 (57.9%) of 501 participants, in the age range of 21-35 years, were seronegative for IgG and IgM anti-*T. gondii* antibodies, and susceptible to acquire acute toxoplasmosis, since this age range is the most common childbearing period. Prenatal screening for *T. gondii* infection is now as important as VDRL, HIV, and HBV, HCV screenings, because toxoplasmosis is a preventable disease. Furthermore, even when the primary infection occurs during the pregnancy, early diagnosis and treatment can reduce the frequency and severity of the disease in neonates (27).

*T. gondii* antibody screening tests in France, Australia and Belgium is mandatory in prenatal care (2, 5). The French national program to identify and treat cases of acute toxoplasmosis in pregnant women has reduced the rate and severity of congenital toxoplasmosis (1, 2). National and regional standards specific to prenatal care for pregnant women have not yet been developed in Iran and so in Khuzestan province.

In conclusion, the present study showed that there were high percentages of negative *T. gondii* antibodies in pregnant women. They were susceptible to acquire acute toxoplasmosis and the subsequent congenital toxoplasmosis with poor pregnancy outcomes. Therefore, prenatal *T. gondii* antibody screening tests are recommend in our province after cost-beneficial analysis researches.

There were some limitations for this study:

1. The difference of speaking languages of cases and the questionnaire language led to some difficulties.
2. The avidity test was not available to diagnose acute toxoplasmosis during study.
